# The cost-utility of open prostatectomy compared with active surveillance in early localised prostate cancer

**DOI:** 10.1186/1472-6963-14-163

**Published:** 2014-04-10

**Authors:** Florian Koerber, Raphaela Waidelich, Björn Stollenwerk, Wolf Rogowski

**Affiliations:** 1 Institute for Health Economics and Health Care Management, Helmholtz Zentrum Munich, German Research Center for Environmental Health (GmbH), Ingolstädter Landstrasse 1, 85764 Neuherberg, Germany; 2 Department of Urology, University of Munich, Marchioninistraße 15, 81377 Munich, Germany; 3 Institute and Outpatient Clinic for Occupational, Social and Environmental Medicine, University of Munich, Ziemssenstraße 1, 80336 Munich, Germany

**Keywords:** Economic evaluation, Cost-utility analysis, Cost-effectiveness, Prostate cancer, Active surveillance, Decision analysis, Early evaluation

## Abstract

**Background:**

There is an on-going debate about whether to perform surgery on early stage localised prostate cancer and risk the common long term side effects such as urinary incontinence and erectile dysfunction. Alternatively these patients could be closely monitored and treated only in case of disease progression (active surveillance). The aim of this paper is to develop a decision-analytic model comparing the cost-utility of active surveillance (AS) and radical prostatectomy (PE) for a cohort of 65 year old men with newly diagnosed low risk prostate cancer.

**Methods:**

A Markov model comparing PE and AS over a lifetime horizon was programmed in TreeAge from a German societal perspective. Comparative disease specific mortality was obtained from the Scandinavian Prostate Cancer Group trial. Direct costs were identified via national treatment guidelines and expert interviews covering in-patient, out-patient, medication, aids and remedies as well as out of pocket payments. Utility values were used as factor weights for age specific quality of life values of the German population. Uncertainty was assessed deterministically and probabilistically.

**Results:**

With quality adjustment, AS was the dominant strategy compared with initial treatment. In the base case, it was associated with an additional 0.04 quality adjusted life years (7.60 QALYs vs. 7.56 QALYs) and a cost reduction of €6,883 per patient (2011 prices). Considering only life-years gained, PE was more effective with an incremental cost-effectiveness ratio of €96,420/life year gained. Sensitivity analysis showed that the probability of developing metastases under AS and utility weights under AS are a major sources of uncertainty. A Monte Carlo simulation revealed that AS was more likely to be cost-effective even under very high willingness to pay thresholds.

**Conclusion:**

AS is likely to be a cost-saving treatment strategy for some patients with early stage localised prostate cancer. However, cost-effectiveness is dependent on patients’ valuation of health states. Better predictability of tumour progression and modified reimbursement practice would support widespread use of AS in the context of the German health care system. More research is necessary in order to reliably quantify the health benefits compared with initial treatment and account for patient preferences.

## Background

Prostate cancer (PC) – ICD code C.61 ‘Malignant neoplasm of the prostate’ following ICD-10-GM classification – is the second most frequent cancer among males in economically developed countries and the most common cancer in Germany, accounting for 14% and 25% of total new cancer cases respectively [1]. Since 1990, the number of new cases has risen by over 50%, amounting to more than 80,000 new diagnoses in Germany in 2010 [2]. The increase in PC incidence has been related to improved means of early diagnosis, especially through prostate-specific antigen (PSA) testing [3]. Prostatectomy (PE) is the first line treatment option for early stage PC. PE is considered the gold standard in urology because other options such as radiotherapy (RT) cannot guarantee complete elimination of tumour cells in the prostate [4,5]. It is also the only treatment for which there exists favourable high quality clinical evidence [6,7]. Accordingly, the German Federal Joint Committee (‘Gemeinsamer Bundesausschuss’, GBA) decided that PE is the preferred treatment option for early stage PC in low risk patients because of the lack of prospective, randomised evidence for RT [6].

Because most carcinomas are thought to have a protracted natural history and more than 85% of patients are older than 65 years at the time of diagnosis, most patients die with the disease and not of it [8,9]. This is especially true for carcinomas that exhibit a low risk profile, i.e. a low PSA value, no histological conspicuity suggested by an indicator such as the Gleason score and confinement to the prostate. For such men, the risk of over-treatment is associated with negative health impacts resulting from the adverse effects of prostatectomy [10,11]. Postoperative rates of incontinence (IC) or erectile dysfunction (ED) of 97% and 72%, respectively, have been reported within 90 days of PE [12]. Despite the fact that some patients recover in the long term, these adverse effects (AE) significantly reduce health related quality of life [13].

As a consequence, observing strategies have been proposed as an alternative to initial treatment [11,14]. Watchful waiting (WW) is a strategy from the pre-PSA test era for patients with limited life expectancy. WW implies no intention to initiate curative treatment. In case of symptomatic disease progression, only palliative treatment is offered to patients, and a survival benefit of primary treatment with PE over WW has been documented in a prospective, randomised controlled trial (RCT) [7,15]. Active surveillance (AS), on the other hand, describes a policy of close monitoring for patients with a life expectancy >15 years. In cases of disease progression, curative treatment is triggered.

There exists no evidence from RCTs for AS [16]. Because AS implies close monitoring and curative treatment when necessary, it can reasonably be assumed that an AS strategy is more effective in avoiding PC specific death than WW. In fact, some evidence suggests that there is no difference in PC death to be expected between AS and PE [17]. The aim of this article was to develop a Markov model for the evaluation of AS as an alternative strategy to PE for the treatment of early stage, localised prostate cancer in the context of the German health care system. Owing to the lack of evidence for AS, we had to base our analysis on reasonable assumptions which we then challenged using extensive sensitivity analyses.

## Methods

### Evaluation

A decision analytic cost-utility model was developed following the standard of the CHEERS checklist, a general guideline on decision-analytic modelling [18]. It was performed from the perspective of the citizens insured by German Statutory Health Insurance (SHI), which is recommended by the German Institute for Quality and Efficiency in Health Care (IQWIG) and includes costs for SHI and out of pocket payments [19]. The study population consisted of men newly diagnosed with low risk PC, no other severe comorbidities and a life expectancy of >15 years. Low risk PC is characterised by a PSA value ≤10 ng/ml, Gleason score ≤6 and tumour stage ≤T2a [11].

Men enter the model at the age of 65 years, which corresponds to the mean age of the cohort in the underlying clinical study. A Markov model was chosen to represent this cohort’s course of disease through different states over time. Quarter-yearly transition cycles were assumed because significant changes in tumour states could occur after 90 days and long term adverse effects could be apparent. In order to capture the full range of costs and effects, we applied a lifetime horizon of 35 years, assuming an age limit of 100 years.

Health outcomes were measured in quality adjusted life years (QALYs), as quality of life is a central aspect in the decision whether to treat or not. All costs (€) were adjusted to 2011 values. Both health outcomes and costs were discounted by 3%, and the half-cycle correction was applied. The model was implemented in TreeAge Pro 2012.

### Interventions/model structure

**Figure 1:**
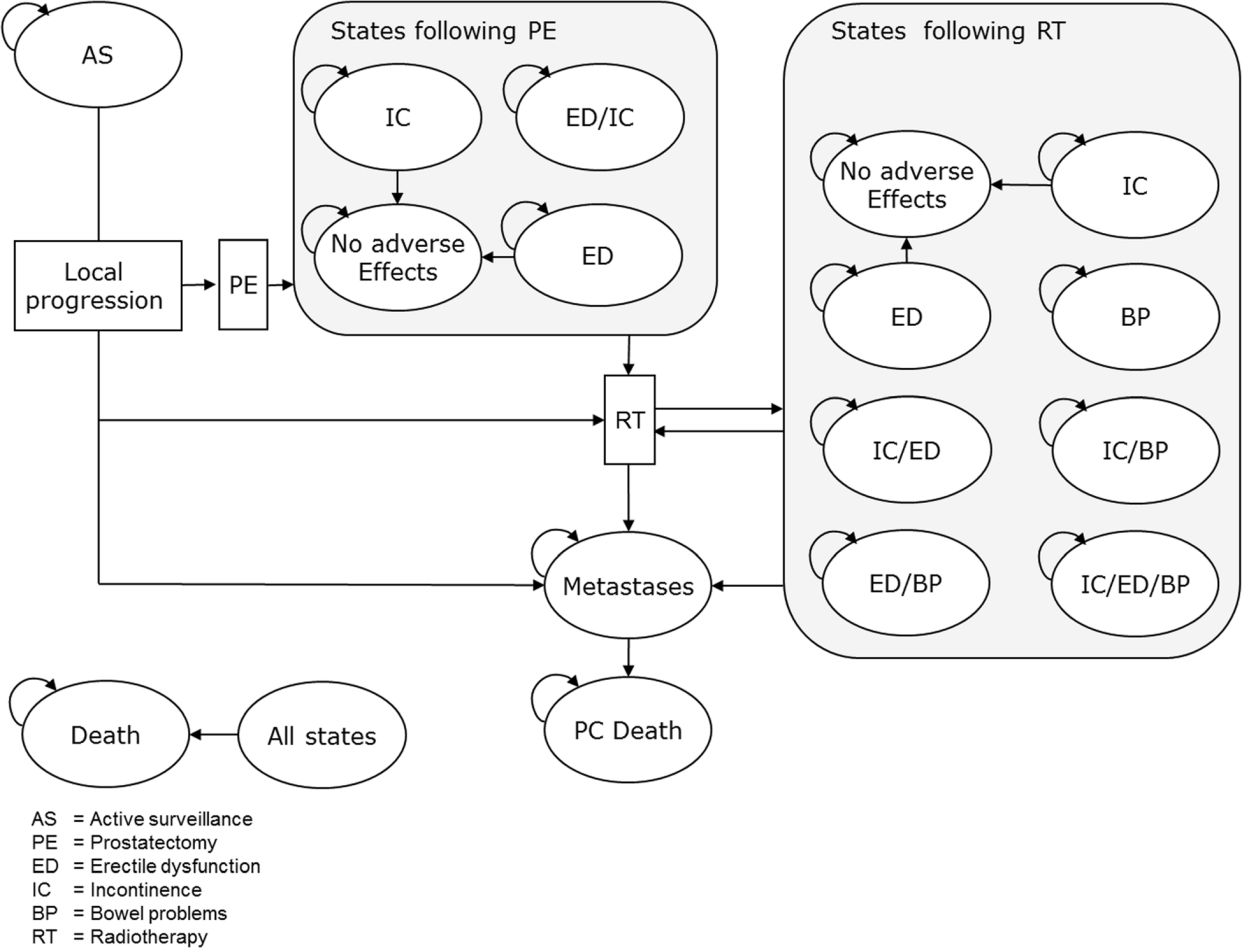
Structure of the model.

The German Association for Urology has published guidelines for the treatment of PC that include AS. According to these, AS involves 3-monthly determinations of PSA value and digital rectal examinations (DRE) in the first 2 years after diagnosis and bi-annually thereafter [5]. Additionally, a biopsy should be taken in the first year and every 3 years after. Treatment can be triggered by an indication of local progression through any of these parameters as well as patient choice. Patients aged ≤72 years are treated by open radical PE; older patients receive RT. A recent review revealed no RCTs comparing the effectiveness of RT and PE with respect to PC mortality [20]. Conservatively, it was assumed that RT and PE have the same disease related outcomes. Downstream treatments such as treatment of adverse effects, prostate hyperplasia and advanced disease were assumed not to have an influence on the difference in mortality between AS and PE. Despite close monitoring, rapidly growing tumours might progress unnoticed under AS and develop metastases prior to treatment [21,22]. Complications occurring within 30 days of PE include rectal injury, wound infection, haemorrhage requiring blood transfusion, deep vein thrombosis and myocardial infarctions [23]. Short term adverse effects such as ED and/or IC are characterised by occurrence and resolution within 90 days after surgery. Long term adverse effects persist after 90 days and can be cured only by surgical intervention. In cases of local recurrence after initial PE, RT is the primary treatment option [5]. As with PE, the adverse effects of RT can be divided into short term and long term effects. In addition to IC and ED, bowel problems (BP) such as abdominal pain, bloating and diarrhoea may develop [24]. Local recurrence is a prerequisite for developing metastatic disease after initial treatment. Once metastases have developed, there is no chance of cure and patients will eventually die of prostate cancer (Figure 1) [25].

### Utilities

#### Baseline utilities

**Figure 2:**
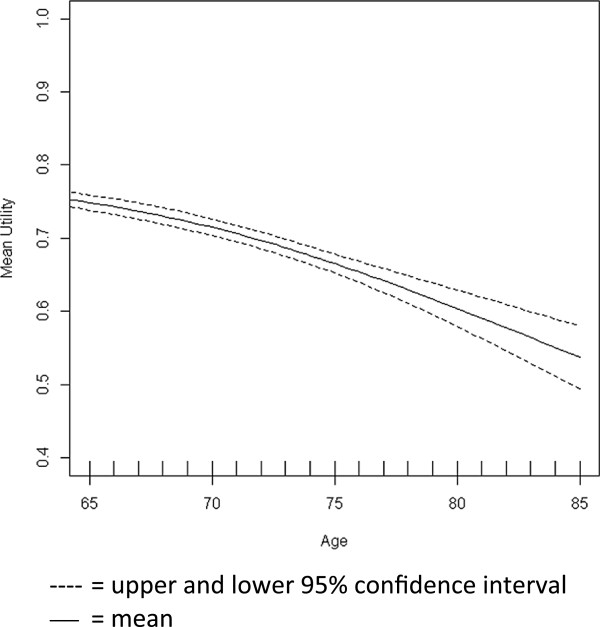
Age adjusted baseline utility German population.

Age-adjusted utility values from the general population provide a reasonable approximation when condition specific baseline data are not available [26]. Health state specific utilities were thus applied as a multiplicative factor to average, age-adjusted utilities from the German male population. The latter are based on a representative study among German citizens (n = 2,049) surveying the EQ-5D items in the years 2006-2011 [27]. Based on these data, the functional relationship between mean EQ-5D utilities and age was estimated with a generalised additive regression model using cubic regression splines (Figure 2) [25].

#### Health state specific utilities

We identified five studies that reported utility weights for relevant health states. Two of these presented implausible or inconsistent results because combined adverse effects were valued more highly than single ones or utility weights >1 were possible, respectively [28,29]. One recent study reported values depending on age and socio-economic status, which could not be adequately combined with our baseline utilities [30]. Stewart et al. provide mean utilities for postoperative health states from a cohort of 162 men [31]. These values compare well with the results of Sommers et al. [32]. Stewart et al. additionally reported utility values for treatment states and combined adverse events, such as ED and IC. Utility values for combined adverse events were surveyed as separate health states so no combination method had to be applied. Furthermore, the quality of life effects of conservative, i.e. non-surgical, downstream treatments such as incontinence pads were already incorporated in the description of health states. We therefore decided to use their preference-based set of utilities elicited by the standard gamble method. Following Liu et al. and the results of the meta-analysis by Bremner et al., we assumed that life under AS has the same utility as life after treatment without side effects [28,33] (Table 1).

**Table 1:** Utility weights of relevant health states

**State**	**Expected value**	**SE**	**95% CI**	**Source**
During AS	0.99	0.05	1; 0.9	Bremner [28], Liu [33], own calculation
Urinary difficulty during AS	0.89	0.024	0.91; 0.85	Steward 2005
During PE treatment	0.67	0.041	0.75; 0.59	Steward 2005
During radiotherapy	0.73	0.045	0.82; 0.64	Steward 2005
Post treatment no adverse effects	0.99	0.05	1; 0.9	Bremner [28], Liu [33], own calculation
Post treatment IC	0.83	0.022	0.87; 0.79	Steward 2005
Post treatment ED	0.89	0.013	0.92; 0.86	Steward 2005
Post treatment BP	0.71	0.021	0.75; 0.67	Steward 2005
Post treatment IC, ED	0.79	0.033	0.86; 0.72	Steward 2005
Post treatment IC, BP	0.70	0.036	0.77; 0.63	Steward 2005
Post treatment ED, BP	0.57	0.039	0.65; 0.49	Steward 2005
Post treatment IC, ED, BP	0.45	0.044	0.54; 0.36	Steward 2005
Metastatic disease	0.25	0.015	0.28; 0.22	Steward 2005

AS = Active surveillance.

PE = Prostatectomy.

IC = Incontinence.

ED = Erectile Dysfunction.

BP = Bowel Problems.

### Costs

Following the perspective of citizens insured by German SHI, all direct medical costs incurred by the SHI as well as by individual patients were included [19]. Indirect costs were neglected as the study population has passed retirement age. Equally, post hospital rehabilitation was not considered as it is typically covered by pension funds. Resource usage was identified and quantified through literature research and treatment recommendations from the Association of German Urologists. Out-patient unit prices are based on the physician’s fee catalogue 2011 (0.035048 cents/point) [34]. In-patient unit prices are based on diagnosis related group (DRG) weights from the German DRG catalogue and the federal base rate for 2011 of €2,963 [35]. For the pricing of pharmaceuticals, we referred to the German formulary 2011 [36]. Remedies and other aids were valued according to market prices investigated using internet research as well as telephone interviews.

#### Primary treatments

In the German DRG system, re-hospitalisations within 30 days are coded as one case. Hence, the costs of PE with and without complications are reflected by the respective DRGs (Table 2). Postoperative monitoring takes place on an out-patient basis (Table 3). Physicians can bring to account a maximum of four patient visits per annum. AS implies determination of PSA values, DRE and regular biopsies (Table 4). Despite preventive antibiotics, biopsy may cause urosepsis which requires hospitalization [37]. Furthermore, symptoms of benign prostate hyperplasia can develop in patients under AS. We assumed that initially half these patients are treated with alpha-1 adrenergic antagonists (Tamsulosin) and the other half with 5-alpha-reductase inhibitors (Finasteride). Patients experiencing worsening of symptoms of urinary difficulty require surgical intervention with transurethral resection of the prostate (TURP) (Table 5). RT is undertaken by a specialist practitioner. Curative treatment entails two target volumes with a maximum of 72 gray, which is equivalent to 40 times 1.8 gray (Table 6).

**Table 2:** In-patient costs of prostatectomy

**In-patient treatment**	**DRG**	**Total costs (€)**
Prostatectomy	M01B	6,886
Complications	M01A	9,526

**Table 3:** Out-patient costs of prostatectomy

**Item**	**Quantity p.a.**			**Price/unit (€)**	**Total costs p.a. (€)**		
Follow-up year	*< 2*	*2-4*	*> 4*		*< 2*	*2-4*	*> 4*
PSA value	4	2	1	4.8	19.20	9.60	4.80
Consultation fee	4	2	1	1.75	7.00	3.5	1.75
Treatment fee > 60 yrs	4	2	1	21.20	84.80	42.40	21.20
Insuree lump sum	4	2	1	9.11	36.45	18.22	9.11

PSA = Prostate Specific Antigen.

**Table 4:** Out-patient costs of active surveillance

**Item**	**Quantity p.a.**			**Price/unit (€)**	**Total costs p.a. (€)**		
AS year	*1*	*2*	*> 2*		*1*	*2*	*> 2*
DRE	4	4	2		Included in lump sum		
PSA value	4	4	2	4.80	19.20	19.20	9.60
Biopsy	1	0.33	0.33	18.58	18.58	6.19	6.19
Consultation fee	4	4	1	1.75	7.01	7.01	1.75
Treatment fee > 60 yrs	4	4	2	21.20	84.82	84.82	42.41
Insuree lump sum	4	4	2	9.11	36.45	36.45	18.22
**Medication**							
Antibiotics (preventive)	0.5	0.16	0.16	16	8.00	2.64	2.64
**Sum**					**174.05**	**156.33**	**80.84**

DRE = Digital Rectal Examination.

PSA = Prostate Specific Antigen.

**Table 5:** Costs other

**Item**	**DRG**	**Quantity p.a.**	**Price/unit (€)**	**Total costs p.a. (€)**
**In-patient treatment**				
**Surgical**				
Prosthesis*	M03C, ZE 58	1	10238.03	
Sphincter*	M01B, ZE 10	0.5	6393.77	3196.14
Sling*	L06A, ZE139	0.5	3677.58	1388.79
Treatment of urosepsis	T60E	1	3075.59	
TURP	M02A	1	3768.93	
**Out-patient treatment**				
**BPS medication**				
Finasteride (5 mg, N3)		1.825	139.88	255.28
Tamsulosin (0.4 mg, N3)		1.825	96.43	175.98

*not covered by statutory health insurance (out of pocket).

BPS = Benign prostate syndrome.

TURP = Transurethral resection of the prostate.

**Table 6:** Out-patient costs of radiotherapy

**Item**	EBM*	**Quantity p.a**	**Price/unit (€)**	**Total costs p.a. (€)**
Consultation fee	25011	1	61.86	61.86
CT Planning	34360	1	38.38	38.38
Radiation plans	25342	2	247.44	247.44
Lump sum/radiation field	40840	15	140	2100
Radiation	25321	40	35.22	1408.93
>2 fields	25322	40	6.48	259.36
3D-planning	25232	40	9.46	378.52
**Sum**				**4741.92**

*EBM = ‘Einheitlicher Bewertungsmaßstab’, i.e. position in the catalogue of reimbursed out-patient services.

#### Adverse effects

The numbers of general practitioner (GP) and specialist practitioner (SP) consultations due to diagnosis of erectile dysfunction were derived from a costing study by Wilson et al. [38]. We estimated consumption of remedies and aids based on the assumption that 70% of patients would make use of phosphodiesterase (PDE) inhibitor and 10% of cavernous injections, SKAT/MUSE or a vacuum pump respectively (Table 7). Symptomatic treatment of IC is achieved through the use of pads in the majority of patients (90%). We assumed an equal distribution of strong, medium and low pads and an average use of three pads/day. Diapers or permanent catheters are necessary in 5% of all patients (Table 8). Costs of managing BP were based on a publication by Hummel et al. [20].

**Table 7:** Costs of managing erectile dysfunction

**Item**	**EBM**	**Quantity p.a**	**Price/unit (€)**	**Total costs p.a. (€)**
**Treatment of symptoms**				
**Out-patient**				
Specialist practitioner				
Consultation fee	1436	1	1.75	1.75
Treatment fee > 60 yrs	26212	1	21.20	21.20
Insuree lump sum	1320	1	9.11	9.11
General practitioner				
Consultation fee	1436	2	1.75	3.50
Treatment fee > 60 yrs	3112	2	35.75	71.50
Insuree lump sum	3111	2	15.77	31.54
Remedies and aids*				
Sildenafil		8.75	44	385
Cavernous injection		2.5	36.62	91.55
(SKAT, MUSE)		2.5	33.19	82.98
Vacuum pump		0.05	301.76	2.66
Ring		4	17	68.00
**Sum**				**768.80**

*not covered by statutory health insurance (out of pocket).

**Table 8:** Costs of managing incontinence

**Item**	**Quantity p.a**	**Price/unit (€)**	**Total costs p.a. (€)**
**Treatment of symptoms**			
**Out-patient**			
Specialist practitioner			
Consultation fee	1	1.75	1.75
Treatment fee > 60 yrs	1	21.20	21.20
Insuree lump sum	1	9.11	9.11
General practitioner			
Consultation fee	2	1.75	3.50
Treatment fee > 60 yrs	2	35.75	71.50
Insuree lump sum	2	15.77	31.54
**Remedies and aids**			
Pads	983	0.36	350.53
Diapers (20 × 20)	19	0.56	10.3
Net trousers for pads/diapers	0.95	10	9.5
Physiotherapy (Pelvic floor)	12	15	180
Balloon catheter	0.6	21.18	12.7
Bed bag sterile	6.1	2.51	15.3
Leg bag sterile	6.1	4.51	27.4
**Sum**			**744.34**

#### Metastases

Metastatic stage is characterised by two phases. At first, cancer is responsive to treatment with luteinizing hormone-releasing hormone (LHRH) agonists which delay progression. Following treatment guidelines, we assumed a dose of 11.5 mg every 3 months. Eventually, patients will become refractory and require chemotherapy. Chemotherapy implies treatment with 142.5 mg of Docetaxel and 5 mg of Prednisolone every 3 weeks. Additionally, around 70% of all refractory patients will develop bone metastases which are treated with zoledronic acid and RT (Table 9) [39]. Radiation therapy assumes a target volume of 35 gray, i.e. 14 times 2.5 gray.

**Table 9:** Costs of managing metastatic disease

	**Quantity p.a**	**Price/unit (€)**	**Total costs p.a. (€)**
**Responsive**			
**Out-patient**			
Specialist practitioner	4	See above	128.28
**Medication**			
LHRH Agonist leuprorelin	4	415	1,660
**Refractory**			
**Medication**			
Docetaxel	17.3	1768	30,645.3
Prednisolon	0.87	10.6	9.2
**Bone metastases**			
**Out-patient**			
Radiation	14	See Table 5	1,484.06
**In-patient**			
Bone scan	0.7	1629.65	1,140.76
**Medication**			
Zoledron acid	12	367.98	4,415.76

LHRH = Luteinizing Hormone-Releasing Hormone.

### Probabilities

#### Mortality

No RCTs comparing disease related mortality of PE and AS could be found in the literature [16]. One American and one Scandinavian RCT were identified comparing WW and initial treatment [7,40]. The 10-year results of the American study (PIVOT) reported overall PC death of 5.8% and 8.4% in the PE and observation arms respectively [40]. This corresponds to a relative risk (RR) of 0.69 which is more favourable towards WW than the results of the Scandinavian Prostate Cancer Group (SPCG). The difference in results is likely to be because the PIVOT cohort represented a population with less advanced disease [40]. However the PIVOT sample also included a large number of African Americans (>30%) who have been shown to suffer from an increased risk of developing and dying from PC [41,42]. In order to avoid country specific bias, we chose to use the SPCG data, which represent the European population more realistically. The SPCG trial found that PE significantly reduced the risk of PC death 15 years after diagnosis with a RR = 0.62, 95% confidence interval (CI) 0.44, 0.87. RR over the time period was estimated by the authors using Cox proportional hazard models. However, the study population (n = 695, mean age 64.7 years) included men with more advanced disease, i.e. PSA value <50, tumour stage ≤ T2 and Gleason score ≤10. Furthermore, patients in the WW group were only treated palliatively in case of disease progression [43]. Following Pearson et al. in the base case, we thus assumed that only half the treatment benefit of PE would be maintained when compared with AS corresponding to a RR of 0.81. This also makes our base case results comparable to the study by Hayes et al. who assume that AS would be 25% more effective than WW, implying a RR of 0.82. We calibrated the transition probability of developing metastases prior to treatment under AS on the basis of the RR of PC mortality after 15 years of 0.81 and the other model parameters. This was based on the assumptions that the additional risk of PC death under AS is constituted by silent progression to metastatic disease and that metastatic PC is a state of terminal illness [4,22,44]. Background mortality was based on the life table of the German Federal Statistical Office 2011 [2].

#### State transition probabilities

We identified a recent systematic review and meta-analysis of studies comparing the benefits and harms of AS and PE for the population in question as best available evidence [45]. If necessary, annual probabilities were translated into quarter-yearly probabilities via conversion to rates [46]. Transition probabilities from short to long term AE could be calculated from the quotient of the probabilities of long term AE and short term AE, i.e. P(transition short term to long term AE) = P(AE long term)/P(AE short term). For transitions to states with combined AE, statistical independence was presumed except in the case of IC. Here, it was assumed that 80% of patients experiencing IC would also experience ED. For an overview of transition probabilities, please refer to Table 10.

**Table 10:** Transition probabilities

**State**	**Event**	**Expected value**	**SE**	Source*
AS	Progression of Gleason Score	0.0263	0.007	
	Other Progression (DRE/PSA)	0.0268	0.007	
	Choosing treatment	0.018	0.005	
	Developing metastatic prostate cancer under active surveillance	0.0023	0.000425	Bill-Axelson [7]; own calculation
	Infection due to biopsy	0.02	0.0075	Cambell-Walsh Urology
	Develop benign prostate hyperplasia	Age dependent		Andersson 2004 [48]
	Transurethral resection of the prostate due to benign prostate hyperplasia	0.000462	-	Andersson 2004; own calculation
Treatment	Perioperative death	0.0044	0.00001	
	Major complication during surgery	0.0472	0.0168	
	Urethral stricture	0.0344	0.002	
Post PE	Incontinence and erectile dysfunction short term	0.37	0.0467	
	Erectile dysfunction short term	0.39	0.0384	
	Incontinence short term	0.09	0.0113	
	Keep incontinence and erectile dysfunction long term	0.27	0.0338	
	Keep incontinence long term	0.28	0.035	
	Keep erectile dysfunction long term	0.89	0.0831	
	Disease recurrence	0.00875	0.0032	
	Progression from recurrence to metastatic disease	0.0127	0.0047	Horwitz 2005 [49]
	Death due to prostate cancer after development of metastatic state during hormonal therapy	0.022	0.0225	Alibhai [44]
Post RT	Incontinence short term	0.3	0.0835	
	Bowel problems short term	0.18	0.0506	
	Bowel problems and incontinence short term	0.054	0.0068	
	Keep incontinence long term	0.16	0.02	
	Keep bowel problems long term	0.152	0.019	
	Erectile dysfunction long term	0.064	0.016	
	Keep incontinence and bowel problems long term	0.148	0.0148	
IC	Sphincter/sling surgery	0.05	0.0075	
ED	Prosthesis surgery	0.02	0.0003	
Death	Death due to other reasons	Age dependent	-	

*If not stated otherwise: [45].

AS = Active Surveillance, ED = Erectile Dysfunction.

DRE = Digital Rectal Examination, IC = Incontinence.

PSA = Prostate Specific Antigen.

PE = Prostatectomy.

RT = Radiotherapy.

### Analysis

#### Sensitivity analysis

**Figure 3:**
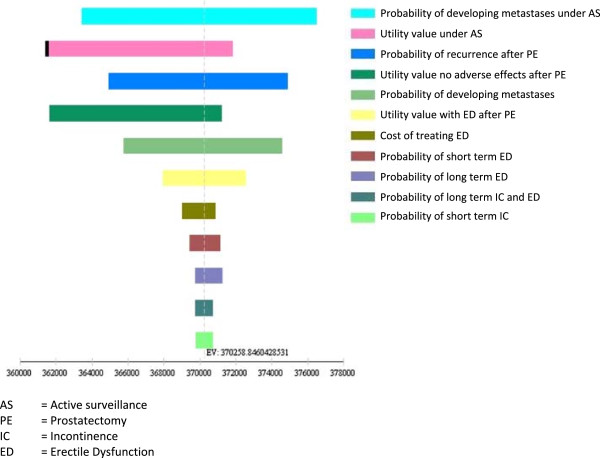
Tornado diagram (Net Benefits in €).

Univariate sensitivity analysis was conducted for all parameters to analyse their isolated impact on cost-effectiveness. For transition probabilities and utilities, input values were varied within the 95% confidence interval. The probability of developing metastases under AS was varied between assuming no difference in disease related mortality compared with PE (i.e. RR =1) and the full treatment effect found in the SPCG trial (i.e. RR = 0.62). Costs for in-patient treatments were varied by running the model with DRG rates resulting from maximum and minimum days of hospitalisation resulting from treatment. All other cost variables were tested by assuming half and double the central estimate. The 10 variables with the highest impact on model uncertainty are presented in a tornado diagram (Figure 3). Given that there exists no explicit cost-effectiveness threshold for Germany, net benefits were calculated with the frequently quoted willingness to pay (WTP) threshold of €50,000, which was chosen for illustration only to incorporate impacts on both effects and costs [49,50]. Based on the tornado analysis, we report threshold values for variables that changed strategy rankings. Key assumptions of the model were varied to test the robustness of the base case results. First, we considered alternative time horizons of 5, 15 and 30 years. Furthermore, we tested the influence of applying alternative discount rates, where both costs and benefits were discounted at the same rate. Following German recommendations, the discount rate was varied between using values of 0%, 5%, 7% and 10% [19,51].

#### Probabilistic analysis

Multivariate probabilistic sensitivity analysis was conducted to assess overall model uncertainty. For this purpose, values were simultaneously and randomly drawn in second order Monte Carlo simulation. Beta distributions were adopted for probabilities and utilities and gamma distributions for costs. The distribution parameters were derived from the model parameter’s expected value and standard error (Tables 1, 10 and 11). In the case of costs, the standard error (SE) was calculated based on the range applied for deterministic sensitivity analysis as follows: SE = (Max value–Min value)/4. The probabilistic sensitivity analysis was based on 1,000 replications, and the results are presented as a cost-effectiveness acceptability curve (CEAC) and as a scatter plot on the cost-effectiveness plane.

**Table 11:** Parameters for probabilistic sensitivity analysis of costs

**Costs item**	Expected value in €*	**SE**
Prostatectomy	6,886	843.75
Conservative treatment of incontinence	186	23.25
Conservative treatment of erectile dysfunction	192	24.03
Radiotherapy	4,742	592.75
Treatment of metastases	447	55.88
Surgical treatment of urosepsis	3,796	384.50
Treatment prostate hyperplasia	108	13.50
Transurethral resection of the prostate	3,769	86.25
Surgical treatment of incontinence	2,292	286.50
Surgical treatment of erectile dysfunction	10,238	1,279.75
Treatment of refractory metastases	7,663.75	957.97
Treatment of bone metastases	1,760.25	220.03

*Quarter yearly except for surgical procedures.

#### Validation

For the sake of cross validation a structured literature search was performed in the databases PubMed, NHS Centre for Reviews and Dissemination as well as Google Scholar to compare our results with existing economic cost-utility models of AS and PE. First we looked for existing reviews of economic evaluations using the (Mesh-) terms ‘review’, ‘prostatic neoplasm’ and ‘economics’. After screening titles and abstracts for the terms ‘model’, ‘evaluation’, ‘cost(s) ’, ‘utility’, ‘quality of life’, ‘effectiveness’ and ‘benefit’ we analysed full texts. In a second step we searched for economic evaluations using the (Mesh-) terms ‘prostatic neoplasm’ and ‘economics’. Face validity of the model structure and major model assumptions was undertaken within our modelling team (FK, BS) and with our clinical expert (RW). Furthermore, we compared model results with clinical data from the American PIVOT trial for the purpose of external validation.

## Results

### Base case

Expected, discounted life expectancy was 12.07 years under AS and 12.15 years with initial surgery if not adjusted for quality of life. This was associated with discounted costs of €16,468 for PE and €9,585 for AS. Treatment with PE therefore generated an additional 0.08 life years and caused additional costs of €6,883, corresponding to an incremental cost-effectiveness ratio (ICER) of €96,420 /life year gained. Some 48% or €7,935 of overall costs were caused by initial treatment in the PE arm. Treatment costs resulting from PE or RT amounted to €3,463 in the AS arm, accounting for 36% of all costs. Costs for AS only amounted to €2,178, making up 22% of total costs. After adjusting for quality of life, effects decreased to 7.60 QALYs under AS and 7.56 QALYs with initial surgery. So AS dominated initial treatment, causing higher effects (+0.04) and lower costs (−€6,883) in the base case. The lifetime risk of PC death was 11.49% under AS and 10.92% in the PE cohort.

### Sensitivity analysis

AS dominated initial surgery in all time perspectives. Because the average health of the population as well as the share of people under AS decreases over time, the benefit of avoiding postoperative AE is most influential in the first years after diagnosis. As the share of people under AS decreases and PC mortality increases, this effect is temporarily compensated for between years 3 and 15. After this, rapidly increasing other cause mortality limits the relative influence of additional PC mortality, which correspondingly puts more weight on patients still under AS. With increasing values for the discount rate, the amount of QALY gains and cost savings decreased, but AS remained the dominant strategy for all discount rates between 0% and 10% (Table 12). Figure 3 depicts the results of the univariate sensitivity analyses in the form of a tornado diagram that displays the effect of the uncertainty associated with individual parameter values on the net monetary benefits of AS for a WTP of €50,000. The utility weight for patients under AS and the probability of developing metastases under AS have the highest impact on model results. Probabilities of recurrence after PE and developing metastases as well as the utility weight for no AE after PE are almost equally influential variables. Threshold analysis revealed that seven of the most influential variables changed the strategy ranking when varied within their 95% confidence intervals (Table 13). The probability of developing metastases under AS proved to be particularly influential. The strategy ranking changed at a threshold value of P = 0.0025 corresponding to a RR of prostate cancer death of 0.76. Additionally we performed a threshold analysis for the proportion of patients under AS crossing over to curative treatment. This proportion is driven by the probability of disease progressing for any reason (i.e. Gleason score or DRE + PSA) and men electing treatment without signs of progression. In the base case this corresponds to an annual crossover probability of P_crossover_ = 0.071 and 61% of patients under AS being treated. PE strategy became more effective than AS at a threshold value of P_crossover_ = 0.149 with 81% of AS patients crossing over to radical treatment.

**Table 12:** Results of sensitivity analysis

**Parameter**	**Value**	**Costs (€)**			**Effects (QALY)**			**ICER (€/QALY)**
		**PE**	**AS**	**Difference**	**PE**	**AS**	**Difference**	
Base case	Time horizon 5	11,355	4,080	−7,275	2.971	3.019	−0.048	Dominated
	Time horizon 15	15,011	8,263	−6,748	6.454	6.467	−0.013	Dominated
	Time horizon 30	16,444	9,564	−6,880	7.545	7.567	−0.022	Dominated
Discount rate	0%	19,013	12,201	−6,811	9.778	9.800	−0.022	Dominated
	5%	15,291	8,346	−6,945	6.525	6.549	−0.025	Dominated
	7%	14,386	7,376	−7,010	5.713	5.739	−0.027	Dominated
	10%	13,376	6,270	−7,106	4.794	4.824	−0.029	Dominated

**Table 13:** Results of threshold analysis

**Variable**	**Base case value**	**Threshold value**
Probability of developing metastases under AS	0.0023	0.0025
Probability of PC recurrence after PE	0.00875	0.00772
Utility value after PE with no adverse effects	0.99	1
Utility value under AS	0.99	0.98
Probability of developing metastases after recurrence	0.0127	0.0113
Utility value after PE with ED	0.89	0.91
Costs of treatment of ED	768.8	None
Probability of short term ED	0.77	0.73
Probability of long term ED	0.89	0.79
Probability of long term ED and IC	0.27	None
Probability of short term IC	0.47	None

AS = Active Surveillance.

PC = Prostate Cancer.

PE = Prostatectomy.

IC = Incontinence.

ED = Erectile Dysfunction.

### Probabilistic sensitivity analysis

**Figure 4:**
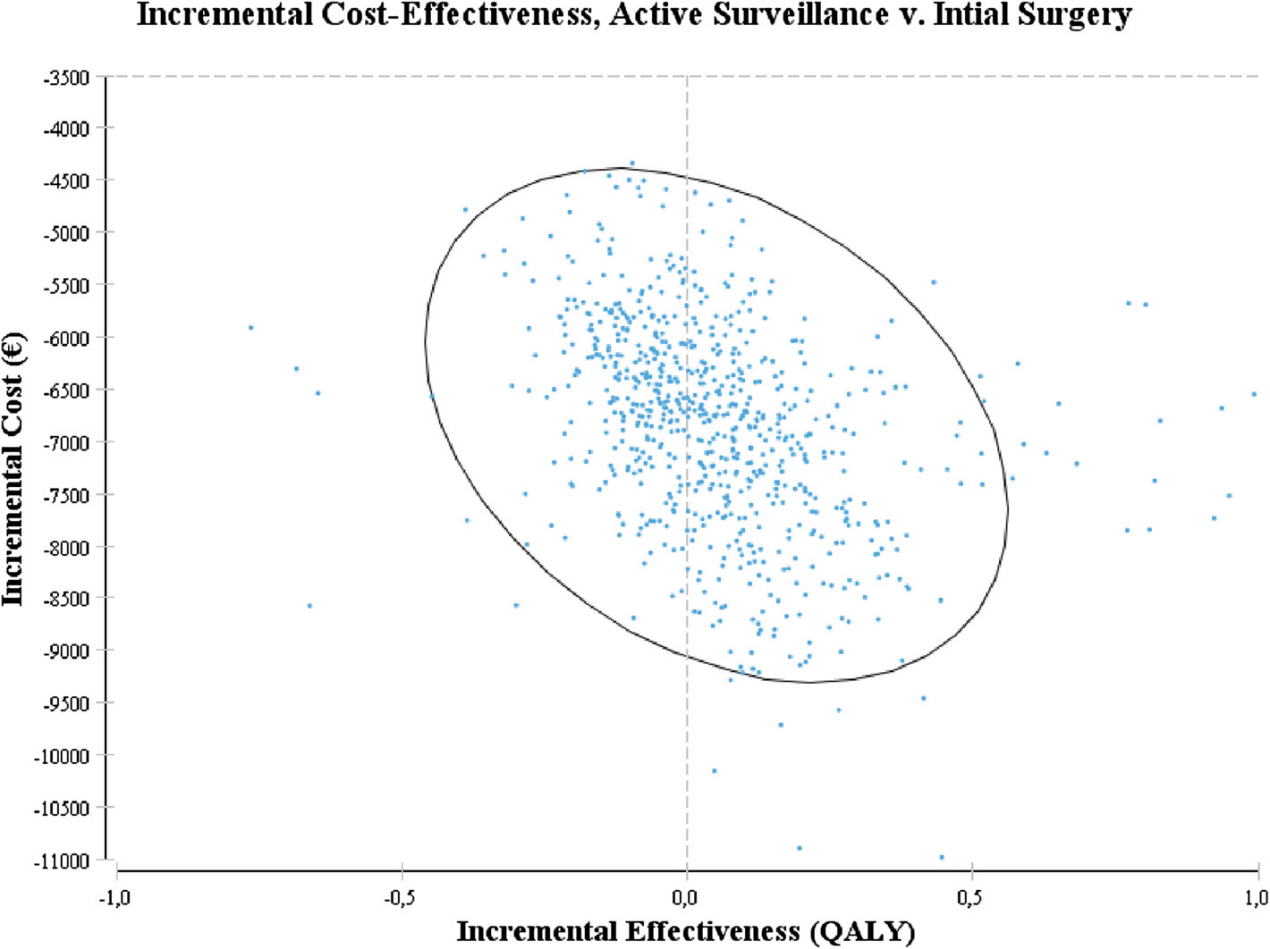
Scatter plot.

**Figure 5:**
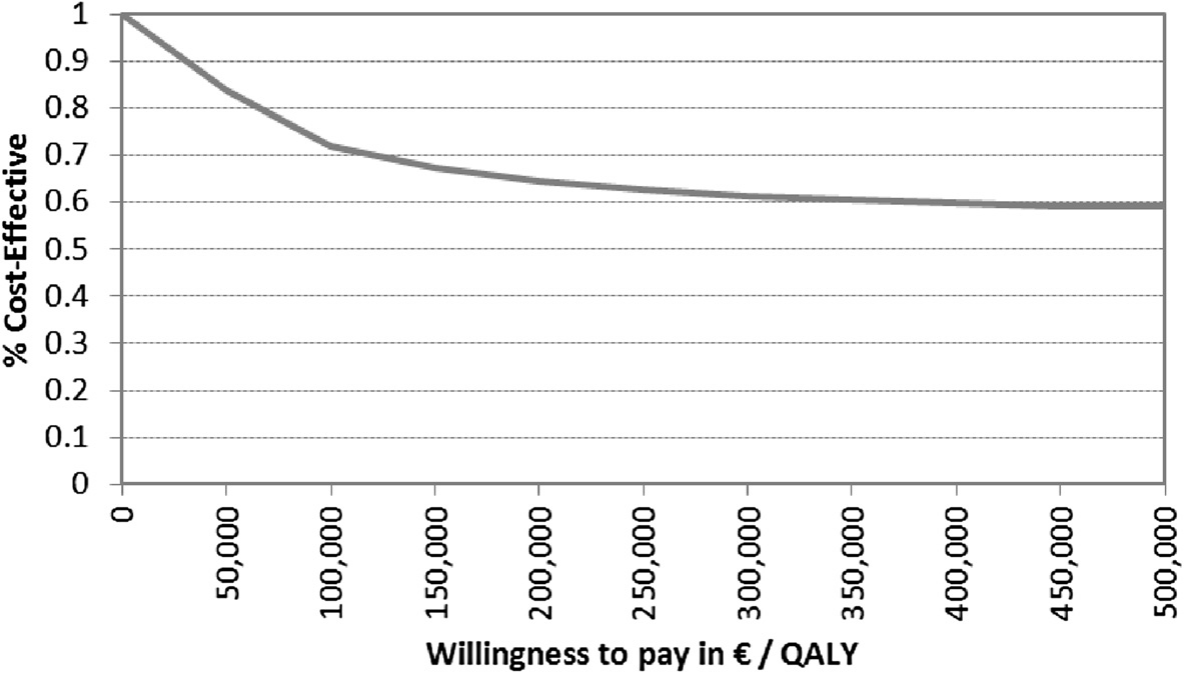
Cost-effectiveness acceptability curve for active surveillance.

Probabilistic analysis resulted in mean discounted costs of €16,415 (95% CI €13,664, €19,339) for PE and €9,564 (95% CI €8,535, €10,735) for the AS strategy. Mean QALYs amounted to 7.58 (95% CI 7.06, 7.93) and 7.60 (95% CI 7.07, 7.83) for PE and AS respectively. Figure 4 shows a scatter plot of ICERs for 1,000 repetitions. AS was the more effective strategy in 56% of all realisations, and it was always associated with lower costs. Figure 5 shows the corresponding CEAC for AS. Even at very high WTP thresholds, the probability of AS being the more effective strategy is more than 50%.

### Validation

Two decision-analytic models could be identified that compared the effectiveness of AS and PE for the treatment of early stage prostate cancer in terms of QALYs generated [29,33], and one other cost-utility study was found [52]. All these studies were undertaken from an American perspective. The models published by Hayes et al. both indicate that more QALYs are generated under AS than with initial PE (11.07 vs. 10.23 and 8.85 vs. 7.95) [29,52]. The study by Liu et al. reports a smaller QALY advantage for a comparable cohort of men. In their study, AS was associated with an additional 0.05 QALYs [33]. The smaller difference in QALYs is likely to be related to the fact that Hayes et al. assume that utility under AS is higher than after PE with no adverse effects. Liu et al. assume equal utility in both states. Our study reports a smaller overall amount of QALYs because the age related decline in quality of life is also considered. The only cost-utility study identified also found AS to be a cost-saving strategy from the perspective of US Medicare [52]. This corresponds to a recent study by Keegan et al. showing that AS is a cost-saving treatment option when compared with immediate treatment in the context of the US healthcare system [53]. Face validation resulted in model adaptations with respect to development and treatment of AEs, length of transition cycles as well as assumptions concerning resource utilisation. For the sake of external validation, we ran the model with the RR of PC death derived from the PIVOT trial. Ceteris paribus this resulted in the same strategy ranking with additional 0.01 QALYs gained in the AS arm (7.61).

## Discussion

We present the first cost-utility study assessing the cost-effectiveness of AS and PE in a European context. Our analysis demonstrated that AS is a cost-saving treatment strategy for men aged 65 years with low risk, early stage carcinoma. AS generates more QALYs at lower costs than treatment with PE in this cohort. The difference in life expectancy was small as other cause mortality accounted for most deaths and limited the influence of treatment specific differences of PC death. Sensitivity to changes in the discount rate and time horizons was low and did not change strategy rankings.

Despite these results, PE is currently widely applied. As the calculation of costs shows, this may be because the current reimbursement rates in Germany set incentives in favour of PE rather than the AS strategy. For example, the restricted ambulatory reimbursement for AS conflicts with the increased patient need for information and counselling. Also, hospitals cannot charge for preventive services and patients are not charged co-payments if they choose the more costly service.

The wide spread of effectiveness results shown in the scatter plot (Figure 4) illustrates that the results are associated with considerable uncertainty surrounding key effectiveness and outcome parameters. Sensitivity analysis revealed that the results are highly sensitive to varying the probability of developing metastases under AS. This reflects the uncertainty concerning the precision of early stage diagnosis and the associated uncertainty in comparative effectiveness between AS and PE. The risk of under-staging, i.e. wrongly diagnosing an aggressive tumour as low risk, due to the limited predictive power of current diagnostic tools is a challenge for current urological research [54]. It has been shown that more than 25% of tumours may be wrongly diagnosed as insignificant in clinical practice [55,56]. Better diagnostic methods for identifying particularly aggressive tumours, e.g. by new molecular markers, analysis of DNA ploidy or CYP3A4 genotype [57], would increase the effectiveness of AS on account of the reduced number of PC deaths due to under staging [54].

Given the currently available staging methods, despite identical clinical parameters, the optimal therapy recommendation may differ depending on the patient’s trade-off between quantity and quality of life and personal risk appetite [32]. Some patients may prefer the avoidance of AEs at the cost of increased risk of dying from PC. Others might not be willing to carry this risk and, at the same time, not consider AEs such as ED as a significant loss of quality of life. For such patients, PE may be a treatment strategy that is considered comparatively expensive but still cost-effective by a number of health care payers. This is highlighted by the fact that, if the lifetime spent in different health states is not adjusted for quality of life, PE is associated with an ICER of €96,420/QALY compared with AS. Also, postoperative rates of IC and ED - the main drivers of QALY advantage under AS–may differ considerably depending on the experience of the surgeon and the overall PE volume of the institution [58,59].

Although consideration of individual patient preference and local setting is an important issue in clinical practice, our study aimed to investigate the cost-utility of AS from a broader health care systems perspective. One of the strengths of our model is the use of age adjusted, population specific utilities in addition to health state specific utility weights. Although this methodology has been demanded by health economists, it is hardly applied in health economic evaluations [60]. Utilities can have a big influence on model results, and disregarding the utility level of the general population overestimates the amount of QALYs generated. Especially in an elderly study population, the effects of age dependent decline in mean utility can significantly influence QALY gains.

Our study is the first evaluation that systematically includes costs for PC management in a European country in the decision analysis. Prices for health services in European countries can differ substantially from those in the US and affect the transferability of results of economic evaluations [61]. The costs quoted for the PSA test in the US evaluation, for example, were almost 80% higher than in Germany, and the costs for PE were over 20% higher (based on an exchange rate of $0.75/€).

### Limitations

A limitation of this study is the restricted quality of evidence concerning disease specific outcomes of treatments. We based our study on the RR of dying from PC from an RCT comparing WW with PE. WW describes a different strategy from AS and is more likely to favour PE as a treatment option. We tried to take account of this by conservatively assuming only half the treatment benefit being maintained under AS and performing wide range sensitivity analysis. We did not include all possible treatment options in our model. There is no conclusive comparative evidence available for alternative treatment options such as brachytherapy or intensity modulated radiation therapy [20,62]. Finally, we assumed that surgical treatment of benign prostate syndrome under AS did not affect the probability of disease progression, which might not be realistic. However, as a reduction in the probability of disease progression would favour AS, this corresponds with our conservative modelling approach.

## Conclusion

The model results indicate that the difference in overall health outcomes between AS and PE is small. On average, approximately one month of life is gained by having immediate surgery; when QALYs are considered, about two additional weeks of life spent in perfect health can be gained by choosing AS. Given the cost difference, the cost-utility analysis replaces the clinical ambiguity with a more solid conclusion that AS may offer better value for money, given the assumptions and perspective of this analysis. In conflict with these results, current reimbursement practise in Germany sets incentives in favour of PE rather than the AS strategy. This study may serve as a starting point to analyse the costs and incentives associated with existing reimbursement patterns in comparison with alternative arrangements.

The model results are subject to substantial uncertainty so that they must be handled with caution. This confirms the importance of ongoing clinical studies, such as the HAROW study in Germany [63] and the German RCT PREFERE [64], that will improve the evidence base in future years. The model needs to be updated as soon as new data from these studies are available. Appropriate staging and risk prediction, which allows the differentiation of high and low risk tumours, plays an important role in decisions about the optimal clinical strategy. Therefore, further research is needed to allow for a better stratification of invasive interventions to high risk patients. This cost-utility analysis can be used for early evaluation of the potential impact of different newly evolving diagnostic strategies on the costs and effects of PC management to inform further research and development [65].

This study revealed that whether PE is considered effective depends not only on clinical data but also on patient preferences about the role of quality of life in decision making. Existing evaluations are typically based on estimates of mean utility gains, which are insensitive to this aspect of benefit. Further research is necessary to better determine the appropriate role of preferences in existing evaluation frameworks. Finally, there is a need for further research on decision aids that make such information accessible to PC patients. Traditional approaches to informing the decision have been shown to understate the importance of postoperative AEs [66]. Ideally, these aspects could be combined so that an analysis of existing incentives and the integration of information from improved biomarker based risk prediction, valuations of health states and cost-effectiveness would lead to new models of fully personalised and cost-effective prostate cancer care.

## Competing interests

The authors declare that they have no financial or non-financial competing interests.

## Authors’contributions

FK programmed the model and drafted the manuscript. RW developed the model structure and provided medical expertise concerning various aspect of urologic health care provision. BS provided statistical expertise for parameter synthesis and analysis. WR conceived the study, and participated in its design and coordination. All authors read and approved the final manuscript.

## Pre-publication history

The pre-publication history for this paper can be accessed here:

http://www.biomedcentral.com/1472-6963/14/163/prepub
